# MACC1 Correlates with Tumor Progression and Immune Cell Infiltration of Colon Adenocarcinoma and is Regulated by the lncRNA ZFAS1/miR-642a-5p Axis

**DOI:** 10.1155/2022/8179208

**Published:** 2022-12-12

**Authors:** Bi-Xia Liu, Jing Yang, Chunyan Zeng, Youxiang Chen

**Affiliations:** Department of Gastroenterology, Digestive Disease Hospital, The First Affiliated Hospital of Nanchang University, 17 Yongwaizheng Street, Nanchang, Jiangxi, China

## Abstract

Colon adenocarcinoma (COAD) is the most common pathologic type of colon cancer. Metastasis is responsible for the high mortality rate of patients with COAD. The gene, metastasis-associated in colon cancer 1 (*MACC*1), is a biomarker predictive of both metastatic and metastasis-free survival in patients with colon cancer and other solid tumors. However, the underlying mechanism by which *MACC*1 affect COAD progression and metastasis remains unknown. In this study, we analyzed the expression level and prognostic value of *MACC*1, as well as their correlation, in patients with various types of cancer included in The Cancer Genome Atlas (TCGA) and Genotype-Tissue Expression (GTEx) databases. High *MACC*1 expression was found to be significantly associated with poor prognosis in patients with COAD. Analysis of the potential upstream miRNA of *MACC*1 showed that miR-642a-5p was downregulated in COAD and was negatively correlated with *MACC*1 expression. Analysis of the upstream regulators of miR-642a-5p showed that the long non-coding RNA (lncRNA) ZFAS1was the most likely upstream regulator of miR-642a-5p. In addition, the expression of *MACC*1 correlated positively with tumor immune cell infiltration, as well as with the levels of biomarkers of five kinds of immune cells. In summary, these findings suggest that *MACC*1 contributes to COAD progression and immune cell infiltration via the ZFAS1/miR-642a-5p/*MACC*1 axis.

## 1. Introduction

Colon cancer is the third most common type of cancer [1] and the second most frequent cause of cancer-related deaths worldwide. It is estimated that more than one million people develop colorectal cancer every year [2]. Colon adenocarcinoma (COAD) is the most common type of colon cancer, accounting for approximately 90% of patients with these tumors. Despite advances in diagnosis and treatment, however, prognosis remains poor in patients with colon cancer, especially those with advanced disease, who are prone to recurrence and metastasis. The 5-year relative survival rate of patients with colorectal cancer has been estimated at approximately 65%, ranging from 90% for patients with localized disease to low as 14% for patients with metastatic disease [3]. The identification of therapeutic targets and the development of effective therapeutic agents to prevent the progression of early disease and to improve survival in metastatic colon cancer are therefore urgently needed.

The metastasis-associated in colon cancer 1 (*MACC*1) gene, which is located on chromosome 7p21.1, was first identified in 2009. Genome-wide analysis of genes differentially expressed in primary colon cancer, metastatic tumors and normal tissues has shown that *MACC*1 is an independent prognostic indicator of metastasis-free survival [4, 5]. *MACC*1 plays a key role in the transition from adenoma to carcinoma in mice and humans [6, 7] and has also been identified as a metastatic and prognostic biomarker for other solid tumors, such as hepatocellular carcinoma, lung cancer, and breast cancer [8–14]. In vivo, *MACC*1 increases tumor cell proliferation and motility, supports cell survival, and regulates metabolism, thereby promoting metastasis. *MACC*1 has also been shown to be involved in tumor immunity. For example, *MACC*1 was found to regulate the expression of PDL1 and tumor immunity through the c‐Met/AKT/mTOR pathway in gastric cancer [15]. However, the prognostic value of *MACC*1 in COAD and its correlation with immune cell infiltration into tumors remain incompletely understood.

The present study analyzed the expression levels and evaluated the prognostic value of *MACC*1 in multiple types of human cancers finding that *MACC*1 was over-expressed in COAD, with this increased expression predictive poor clinical features and poor prognosis. The potential carcinogenic mechanisms of *MACC*1 were assessed in COAD, as well as the correlation between *MACC*1 expression level and the degree of immune cell infiltration. The present study also analyzed the relationship between *MACC*1 expression and biomarkers of immune cells in COAD. These findings showed that the ZFAS1/miR-642a-5p axis is a potential upstream pathway of *MACC*1 in COAD. Taken together, these results indicated that *MACC*1 contributes to COAD progression and immune cell infiltration via the ZFAS1/miR-642a-5p/*MACC*1 axis, suggesting that the ZFAS1/miR-642a-5p/*MACC*1 axis may provide a novel therapeutic target and a valuable prognostic indicator for COAD.

## 2. Materials and Methods

### 2.1. Gene Expression Analysis

The Cancer Genome Atlas (TCGA) database (https://portal.gdc.cancer.gov/) is a cancer genomics program designed to identify and classify major oncogenic genomic changes to enhance our understanding of cancer [16]. The Genotype-Tissue Expression Project (GTEx) is a data resource and tissue bank established by the National Institutes of Health Common Fund that can enable determination of the relationship between genetic variation and gene expression in a variety of human tissues [17]. RNA-seq data for *MACC*1 mRNA and relevant clinical data across 33 cancer types and normal tissues in the TCGA and GTEx database were downloaded from the UCSC XENA website (https://xenabrowser.net/datapages/). Data for various tumor cell lines were downloaded from the Cancer Cell Line Encyclopedia (CCLE) database (https://portals.broadinstitute.org/ccle/). RNA-seq data on gene expression in the FPKM format were converted into TPM format and log2 transformed for further analysis. The levels of expression of *MACC*1 in tumors and corresponding normal tissues in the data downloaded from the TCGA and GTEx databases were analyzed, as were the associations between *MACC*1 expression and clinical characteristics in patients with COAD. Differences in expression were analyzed using the Mann-Whitney *U* test, with *p* < 0.05 defined as statistically significant. Results were visualized using the *R* package ggplot2.

### 2.2. GEPIA2 Database Analysis

Gene Expression Profiling Interactive Analysis 2 (GEPIA2; https://gepia2.cancer-pku.cn/#index) is a web-based tool for interactive analysis based on normal and cancer samples from the TCGA and GTEx databases. GEPIA2 offers a series of functions such as differential expression analysis, profile plotting, correlation analysis, survival analysis, detection of similar genes, and dimensionality reduction analysis, all of which may be customized [18]. The expression levels of *MACC*1 and lncRNAs in COAD tissues from the TCGA normal and GTEx databases as controls were analyzed using the “Expression DIY-Box Plot” module of GEPIA2 using the settings, *p*-value cutoff = 0.001, |log2FC (Fold change)| cutoff = 1, and “Match TCGA normal and GTEx data.” Correlations between *MACC*1 expression and immune cell biomarkers in COAD were analyzed in the “Correlation Analysis” mode of GEPIA2, with correlation coefficients calculated using Spearman analysis. The selection criteria were set as |*R*| > 0.1 and *p* < 0.05 for identifying statistically significant.

### 2.3. Analysis of the CPTAC and UALCAN Databases

The Clinical Proteomic Tumor Analysis Consortium (CPTAC, https://proteomics.cancer.gov/programs/cptac) is a centralized repository of publicly available proteomic sequence datasets. CPATC identifies the protein composition and characteristic proteome of each tumor sample by mass spectrometry [19]. UALCAN (https://ualcan.path.uab.edu) is a user-friendly interactive web-portal designed to facilitate analysis of the correlation between gene expression in tumor subgroups and patient survival [20]. UALCAN was used to analyze the expression of *MACC*1 total protein from CPTAC database, by entering “*MACC*1” and selecting the dataset of colon cancer and *MACC*1 proteomic expression profile based on sample types.

### 2.4. Analysis of Diagnostic and Prognostic Value

The diagnostic value of *MACC*1 in COAD was determined using receiver operating characteristic (ROC) curves. The areas under the ROC curves (AUCs) were between 0.5 and 1, with AUCs >0.9 defined as being highly accurate. The relationships between *MACC*1 expression and indicators of patient prognosis, including overall survival (OS), disease-specific survival (DSS), and progression-free interval (PFI), were evaluated in 33 types of cancers, with the results presented as Forest plots and Kaplan-Meier curves. A uniformly standardized pan-cancer dataset from the UCSC XENA database (https://xenabrowser.net/datapages/), with samples from patients followed-up for less than 30 days and those with fewer than 10 samples in a single cancer species excluded. Hazard ratios (HRs) with 95% confidence intervals (CIs) were calculated by univariate regression analyses. Survival rates in patients with different levels of *MACC*1 expression were determined by the Kaplan-Meier method and compared by log-rank tests. The optimal cut-off value for *MACC*1 expression that differentiated patients into subgroups with high and low expression was calculated by the minimum *p*-value approach using the *R* package maxstat (Version 0.7–25). Statistical analyses were performed using the survival package in *R* software (version 3.6.3), with the forestplot and survminer packages used for visualization. A *p* value <0.05 was considered statistically significant.

### 2.5. starBasev2.0 Database Analysis

The starBasev2.0 database (https://starbase.sysu.edu.cn/) allows systematic identification of RNA-RNA and protein-RNA interaction networks [21], which consist of several online prediction programs, such as PITA, RNA22, miRmap, microT, miRanda, PicTar, and TargetScan. The miRNAs acting upstream of *MACC*1 were predicted by the starBasev2.0 database in the miRNA-mRNA panels. Predicted miRNAs appearing simultaneously in more than two programs were considered candidate miRNAs for *MACC*1 and included in subsequent analyses. Correlation analyses for miRNA and *MACC*1, lncRNA and hsa-miR-642a-5p, and lncRNA and *MACC*1 in COAD samples in the starBasev2.0 database were performed in the miRNA-Target CoExpression or RNA-RNA CoExpression panels. The levels of expression of hsa-miR-642a-5p in COAD and normal tissue were also analyzed in the miRNA Differential Expression panels, as well as to predict candidate lncRNAs that could potentially bind to hsa-miR-642a-5p. The miRNA-mRNA and lncRNA-miRNA interactions networks were visualized by Cytoscape (version 3.8.2).

### 2.6. TIMER2.0 Database Analysis

The TIMER2.0 web server (https://timer.cistrome.org/) allows a comprehensive analysis of gene expression and tumor infiltrating immune cells in multiple cancers [22]. The “Immune-Gene” module of this website was used to analyze the correlations between *MACC*1 expression levels and tumor infiltration by immune cells, including *B* cells, CD8^+^*T* cells, CD4^+^*T* cells, Tregs, NK cells, macrophages, neutrophil cells, and dendritic cells, in COAD were selected. The *p*-values and Rho values were obtained via the purity adjustment Spearman's rank test. The correlations were shown as scatter plots, with *p* < 0.05 considered statistically significant.

### 2.7. Statistical Analysis

Statistical analyses were performed using *R* software (V3.6.3), although some of the statistical analyses were performed automatically using the online databases described above. In all statistical analyses, *p* < 0.05 was considered statistically significant for all statistical analyses.

## 3. Results

### 3.1. Expression of *MACC*1 mRNA in Human Cancers

**Figure 1:**
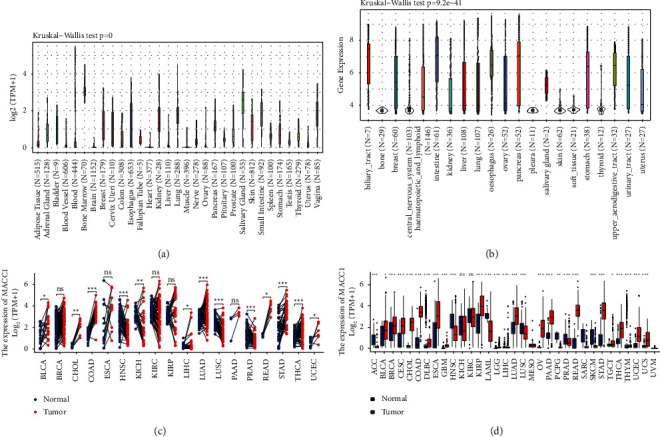
Expression level of *MACC*1 in normal tissues and multiple cancers. (a) *MACC*1 expression in 31 normal tissues based on the GTEx database. (b) *MACC*1 expression in 21 tumor cell lines based on the CCLE database. (c) *MACC*1 expression in 18 paired tumors and adjacent normal tissues based on TCGA database. (d) *MACC*1 expression in 33 TCGA tumors and normal tissues with the data of GTEx database as controls. (ns, *p* ≥ 0.05; ^*∗*^*p* < 0.05; ^*∗∗*^*p* < 0.01; ^*∗∗∗*^*p* < 0.001).

To investigate the potential role of *MACC*1 in carcinogenesis, its level of expression was determined in normal tissues, tumor cell lines and various primary human cancers. Assessment of *MACC*1 expression levels in 31 normal tissues from the GTEx database showed that *MACC*1 was expressed at low levels in most normal tissues, although its expression was higher in bone marrow (Figure 1). Analysis of *MACC*1 expression in 21 tumor cells lines in the CCLE database showed that *MACC*1 was highly expressed in most cell lines (Figure 1(b)). Analysis of the differential expression of *MACC*1 in primary tumors and adjacent normal tissues in data directly obtained from the TCGA database showed that the expression of *MACC*1 was significantly higher in eight types of human cancer, namely bladder urothelial carcinoma (BLCA), cholangiocarcinoma (CHOL), COAD, lung adenocarcinoma (LUAD), rectum adenocarcinoma (READ), stomach adenocarcinoma (STAD), thyroid cancer (THCA), and uterine corpus endometrial carcinoma (UCEC), while significantly lower in five types of cancer, namely head and neck squamous cell carcinoma (HNSC), kidney chromophobe tumors (KICH), liver hepatocellular carcinoma(LIHC), lung squamous cell carcinoma(LUSC), and prostate adenocarcinoma(PRAD) (Figure 1(c)). Because the TCGA database included few normal tissue samples, data on tumor tissues in the TCGA database were combined with data on normal tissues in the GTEx database to analyze the differential expression of *MACC*1 in 33 tumors. Compared with the corresponding normal samples, *MACC*1 expression was significantly higher in 20 types of cancer, namely BLCA, breast cancer (BRCA), cervical squamous cell carcinoma and endocervical adenocarcinoma (CESC), CHOL, COAD, diffuse large *B*-cell lymphoma (DLBC), esophageal carcinoma (ESCA), glioblastoma multiforme(GBM), kidney renal papillary cell carcinoma (KIRP), lower grade glioma (LGG), LUAD, ovarian serous cystadenocarcinoma (OV), pancreatic adenocarcinoma (PAAD), READ, STAD, testicular germ cell tumors (TGCT), THCA, thymoma (THYM), UCEC, and uterine carcinosarcoma (UCS). In contrast, *MACC*1 expression was significantly lower in eight other types of cancer, namely adrenocortical carcinoma (ACC), HNSC, acute myeloid leukemia (LAML), LIHC, LUSC, pheochromocytoma and paraganglioma (PCPG), PRAD, and skin cutaneous melanoma (SKCM). The levels of expression of *MACC*1, however, did not differ significantly in kidney chromophobe (KICH) and kidney renal clear cell carcinoma (KIRC) samples (Figure 1(d)). Taken together, these findings suggest that *MACC*1 may play different roles in the development of different tumors.

### 3.2. The Prognostic Value of *MACC*1 in Human Cancers

**Figure 2:**
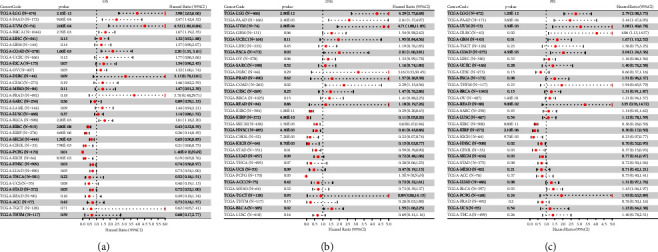
Prognostic value of *MACC*1 in multiple cancers. Forest plot of the relationship of *MACC*1 expression with (a) overall survival (OS), (b) disease-specific survival (DSS) and (c) progression-free interval (PFI) across 33 types of tumors based on TCGA database.

**Figure 3:**
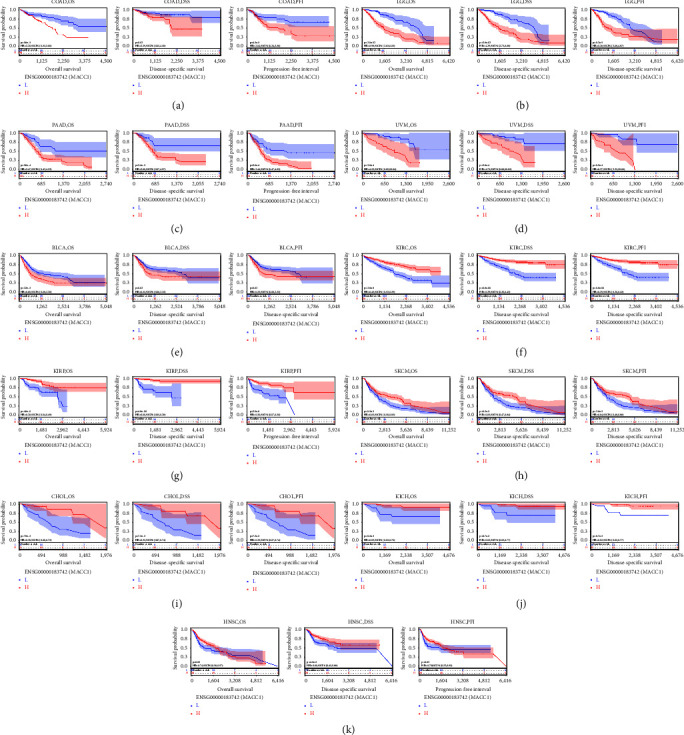
Kaplan-Meier curves of *MACC*1 expression in ten tumors. (a) KM curves of *MACC*1 expression in COAD patients. (b) KM curves of *MACC*1 expression in LGG patients. (c) KM plot of *MACC*1 expression in PAAD patients. (d) KM curves of *MACC*1 expression in UVM patients. (e) KM curves of *MACC*1 expression in BLCA patients. (f) KM curves of *MACC*1 expression in KIRC patients. (g) KM curves of *MACC*1 expression in KIRP patients. (h) KM curves of *MACC*1 expression in SKCM patients. (i) KM curves of *MACC*1 expression in CHOL patients. (j) KM curves of *MACC*1 expression in KICH patients. (k) KM curves of *MACC*1 expression in HNSC patients.

The prognostic value of *MACC*1 expression in patients with 33 types of human cancers from the TCGA database was analyzed. The correlations between *MACC*1 expression with overall survival (OS), disease-specific survival (DSS) and progression-free interval (PFI) were determined using the *R* Package survival (version 3.2–7), with forest plots were used to evaluate the relationship between *MACC*1 expression and patient prognosis. Higher expression of *MACC*1 mRNA was indicative of shorter OS in patients with LGG (HR = 4.05, *p*=8.4*e* − 13), PAAD (HR = 2.50, *p*=8.2*e* − 4), UVM (HR = 4.53, *p*=2.0*e* − 4), BRCA (HR = 1.66, *p*=3.2*e* − 3), COAD (HR = 2.20, *p*=1.6*e* − 3), and BLCA (HR = 1.62, *p*=1.8*e* − 3), but correlated with an increased OS in patients with KIRC (HR = 0.42, *p*=1.2*e* − 8), KIRP (HR = 0.26, *p*=4.6*e* − 6), SKCM (HR = 0.65, *p*=1.5*e* − 3), KICH (HR = 0.19, *p*=8.5*e* − 3), CHOL (HR = 0.27, *p*=0.01), PCPG (HR=1.4*e* − 9, *p*=0.01), and STAD (HR = 0.72, *p*=0.04) (Figure 2). Higher levels of *MACC*1 mRNA expression were associated with shorter DDS in patients with LGG (HR = 4.29, *p*=2.8*e* − 12), PAAD (HR = 2.61, *p*=2.4*e* − 3), UVM (HR = 4.71, *p*=2.8*e* − 4), ESCA (HR = 2.01, *p*=0.03), COAD (HR = 2.29, *p*=0.02), and BLCA (HR = 1.55, *p*=0.02), but were associated with a longer DDS in patients with KIRC (HR = 0.29, *p*=1.8*e* − 11), KIRP (HR = 0.11, *p*=4.8*e* − 10), SKCM (HR = 0.63, *p*=1.5*e* − 3), HNSC (HR = 0.61, *p*=4.3*e* − 3), CHOL (HR = 0.23, *p*=7.2*e* − 3), KICH (HR = 0.15, *p*=8.7*e* − 3), and STAD (HR = 0.59, *p*=0.01) (Figure 2(b)). Higher expression of *MACC*1 mRNA was also associated with a shorter PFI in patients with LGG (HR = 2.79, *p*=1.2*e* − 12), PAAD (HR = 2.44, *p*=3.6*e* − 4), UVM (HR = 3.08, *p*=3.3*e* − 3), DLBC (HR = 4.06, *p*=0.02), GBM (HR = 1.67, *p*=0.01), COAD (HR = 2.04, *p*=4.3*e* − 3), and BLCA (HR = 1.43, *p*=0.03), but correlated with a longer PFI in patients with KIRC (HR = 0.36, *p*=8.8*e* − 11), KIRP (HR = 0.30, *p*=2.1*e* − 6), KICH (HR = 0.23, *p*=9.7*e* − 3), HNSC (HR = 0.70, *p*=0.02), CHOL (HR = 0.37, *p*=0.03), and SKCM (HR = 0.77, *p*=0.03) (Figure 2(c)). The levels of *MACC*1 expression, however, did not correlate with survival parameters in patients with other types of cancer. Taken together, these results suggested that the potential prognostic value of *MACC*1 mRNAs differs among different types of cancer. Moreover, Kaplan-Meier analyses showed that *MACC*1 was significantly correlated with OS, DSS, and PFI in patients with COAD, LGG, PAAD, UVM, BLCA, KIRC, KIRP, SKCM, KICH, and CHOL, suggesting that *MACC*1 expression may be a biomarker in these tumors (Figure 3).

### 3.3. Expression of *MACC*1 in Patients with COAD

**Figure 4:**
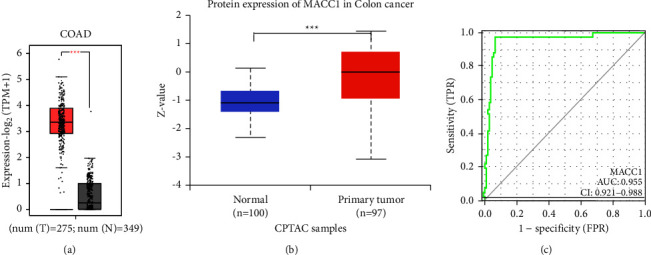
The mRNA, protein expression and ROC curve of *MACC*1 in COAD. (a) The mRNA expression levels of *MACC*1 in COAD tissues and the corresponding normal tissues determined by GEPIA2 database. (b) The protein expression levels of *MACC*1 based on CPTAC database. (c) ROC curve showed that *MACC*1 had an AUC value of 0.955 to discriminate COAD tissues from healthy controls. With a cut-off of 2.756, the sensitivity, specificity and accuracy were 0.976, 0.933, and 0.909, respectively (^*∗∗∗*^*p* < 0.001).

**Figure 5:**
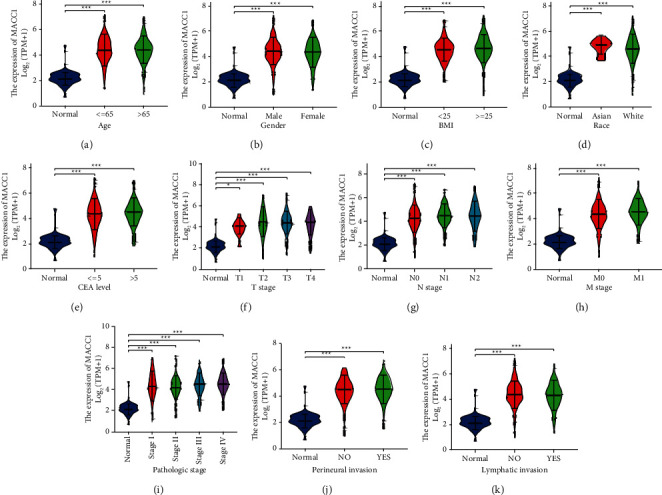
Associations between *MACC*1 expression and different clinical characteristics in COAD. (a) Age; (b) gender; (c) BMI; (d) race; (e) CEA level; (f) *T* stage; (g) *N* stage; (h) *M* stage; (i) pathologic stage; (j) perineural invasion; (k) lymphatic invasion. ^*∗*^*p* < 0.05; ^*∗∗*^*p* < 0.01; ^*∗∗∗*^*p* < 0.001.

Pan-cancer analysis showed that *MACC*1 mRNA was upregulated in COAD. To further understand the importance of *MACC*1mRNA and protein levels in COAD, *MACC*1 expression data from the TCGA and CPTAC databases were analyzed. Unpaired analysis of samples from the GEPIA2 database showed that *MACC*1 mRNA levels were significantly higher in 275 COAD samples than in 349 normal colon tissue samples (Figure 4). Analysis of data from CPTAC in the UALCAN database showed that *MACC*1protein expression was significantly higher in COAD than in normal colon tissue samples (Figure 4(b)). ROC curve analysis of the diagnostic value of *MACC*1 in COAD showed that *MACC*1 mRNA had an AUC of 0.955 (95% CI: 0.921–0.988) in distinguishing COAD from control samples. The optimal *MACC*1 mRNA cut-off, 2.756, had a sensitivity of 0.976, a specificity of 0.933 and an accuracy of 0.909, suggesting that *MACC*1 may be a potential biomarker to distinguish COAD from normal tissues (Figure 4(c)). Analysis of *MACC*1 expression in COAD patients sub-grouped by clinical parameters showed that the levels of *MACC*1 mRNA were higher in COAD than in normal colon tissue samples in all patient subgroups based on age, gender, body mass index (BMI), race, carcinoembryonic antigen (CEA) levels, *T* stage, *N* stage, *M* stage, pathologic stage, perineural invasion and lymphatic invasion (Figure 5). Evaluation of the association between *MACC*1 mRNA levels and clinicopathological characteristics of patients with COAD showed that higher *MACC*1 mRNA levels were associated with distant metastasis and high TNM stage (Table 1). These findings suggest that *MACC*1mRNA and protein were both upregulated in COAD and indicate that *MACC*1 may play an important role in the development and progression of COAD.

### 3.4. Predicted Upstream Potential miRNAs of *MACC*1

**Figure 6:**
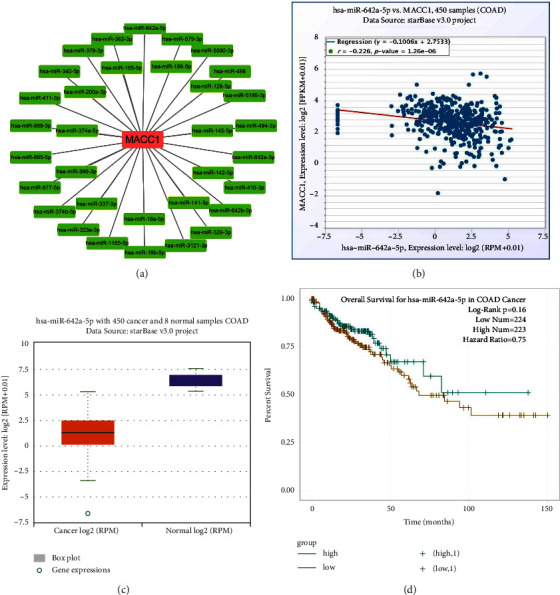
Identified hsa-miR-642a-5p as the most potential upstream miRNA of *MACC*1 in COAD. (a) The miRNA-*MACC*1 network established by cytoscape software. (b) Expression correlation between hsa-miR-642a-5p and *MACC*1 in COAD. (c) Expression levels of hsa-miR-642a-5p in COAD and normal tissues. (d) Prognostic value of hsa-miR-642a-5p in COAD.

Non-coding RNAs (ncRNAs) are an important class of gene regulators responsible for regulating the expression of many key genes at the transcriptional and post-transcriptional levels. miRNAs are mainly involved in the negative regulation of gene expression. To assess whether *MACC*1 is modulated by miRNAs, starBase2.0 was used to predict miRNAs acting upstream of *MACC*1. This analysis identified 33 upstream miRNAs that could potentially bind to *MACC*1 (Supplementary Table S1). To improve visualization, Cytoscape software was used to establish a miRNA-*MACC*1 regulatory network (Figure 6). Because miRNAs usually act by negatively regulating target gene expression, miRNAs were expected to negatively regulate *MACC*1. Evaluation of the correlation between miRNA and *MACC*1 expression showed that *MACC*1 mRNA negatively correlated with the levels of hsa-miR-141-3p, hsa-miR-142-5p, hsa-miR-126-5p, hsa-miR-186-5p, hsa-miR-155-5p, and hsa-miR-642a-5p in COAD (Figure 6(b), Supplementary Table S1). Further determination of the levels of expression of these six miRNAs in COAD showed that hsa-miR-642a-5p was markedly downregulated in COAD (Figure 6(c)) and that lower expression of hsa-miR-642a-5p was associated with poorer OS in patients with COAD (Figure 6(d)). These findings suggest that hsa-miR-642a-5p may regulate *MACC*1 mRNA in COAD.

### 3.5. Predicted Upstream Potential lncRNAs of hsa-miR-642a-5p

**Figure 7:**
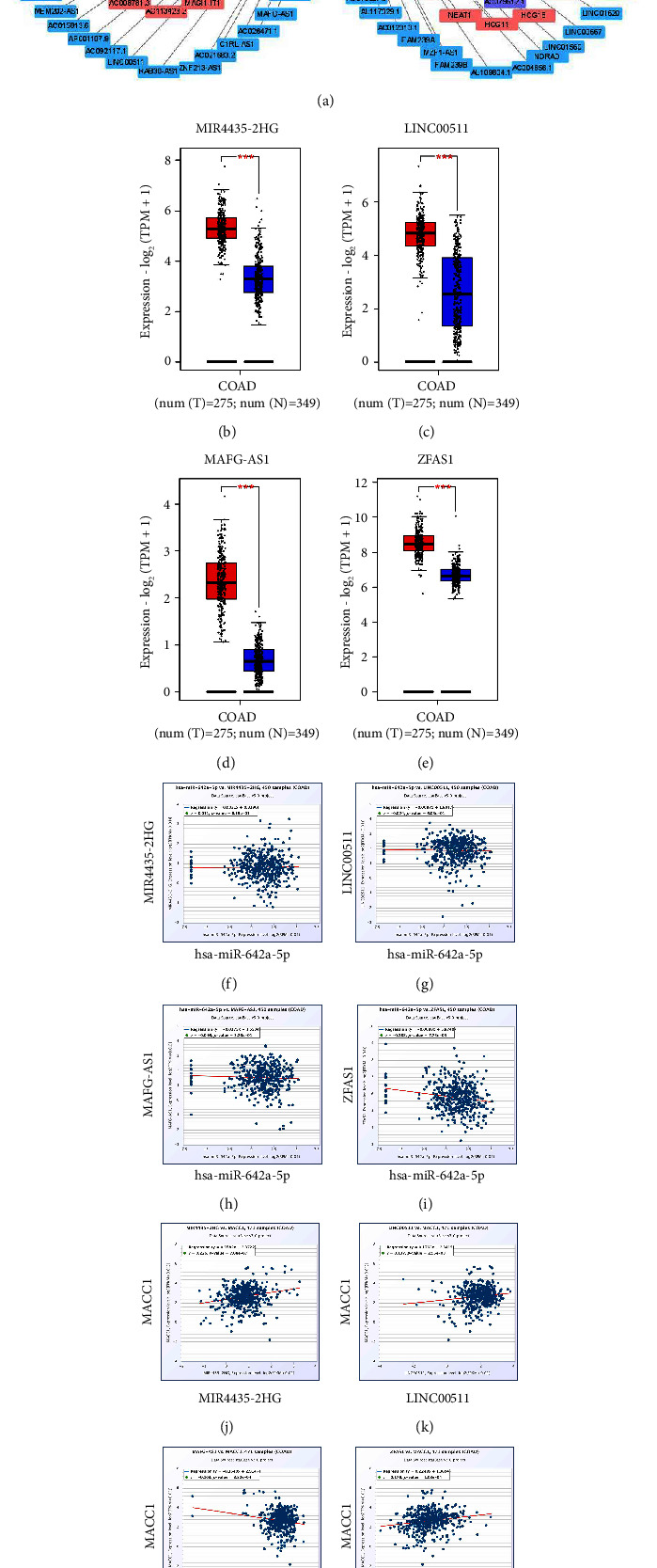
Identification of upstream potential lncRNAs of hsa-miR-642a-5p in COAD determined by starBase database. (a) The lncRNAs-hsa-miR-642a-5p network constructed by cytoscape. (b)–(e) The expression of MIR4435-2HG (b), LINC00511 (c), MAFG-AS1 (d), and ZFAS1 (e) in COAD compared with “TCGA and GTEx normal” data. (f)–(i) The expression correlation between MIR4435-2HG, LINC00511, MAFG-AS1, ZFAS1 and hsa-miR-642a-5p. (j)–(m) The expression correlation between MIR4435-2HG, LINC00511, MAFG-AS1, ZFAS1 and *MACC*1. ^*∗∗∗*^*p* < 0.001.

The starBase database predicted that 113 possible lncRNAs acted upstream of hsa-miR-642a-5p. Cytoscape software was used to improve visualization by constructing a lncRNA-hsa-miR-642a-5p regulatory network (Figure 7). According to the hypothesis of competing endogenous RNA (ceRNA), these lncRNAs should be oncogenes in COAD, with the competitive binding of these lncRNAs to shared miRNAs enhancing the expression of *MACC*1 mRNA. Thus, the levels of these lncRNAs should correlate negatively with the levels of miRNA, but positively with the levels of *MACC*1 mRNA. Determination by GEPIA2 of the levels of expression of the predicted lncRNAs in COAD and corresponding normal tissues showed that, of the 113 possible lncRNAs, only four, MIR4435-2HG, LINC00511, MAFG-AS1, and ZFAS1, were expressed at significantly higher levels in COAD than in corresponding normal tissue samples (Figures 7(b)–7(e)). The correlations of these four lncRNAs (MIR4435-2HG, LINC00511, MAFG-AS1, and ZFAS1) with the levels of hsa-miR-642a-5p and *MACC*1 mRNA in COAD were confirmed using the starBase database. ZFAS1 was negatively correlated with hsa-miR-642a-5p, but positively correlated with *MACC*1 mRNA (Table 2, Figures 7(f)–7(m)), suggesting that ZFAS1 might be the upstream lncRNA of the hsa-miR-642a-5p/*MACC*1 axis in COAD.

### 3.6. Correlation between *MACC*1 Expression with Immune Cell Infiltration and Biomarkers of Immune Cells in COAD

**Figure 8:**
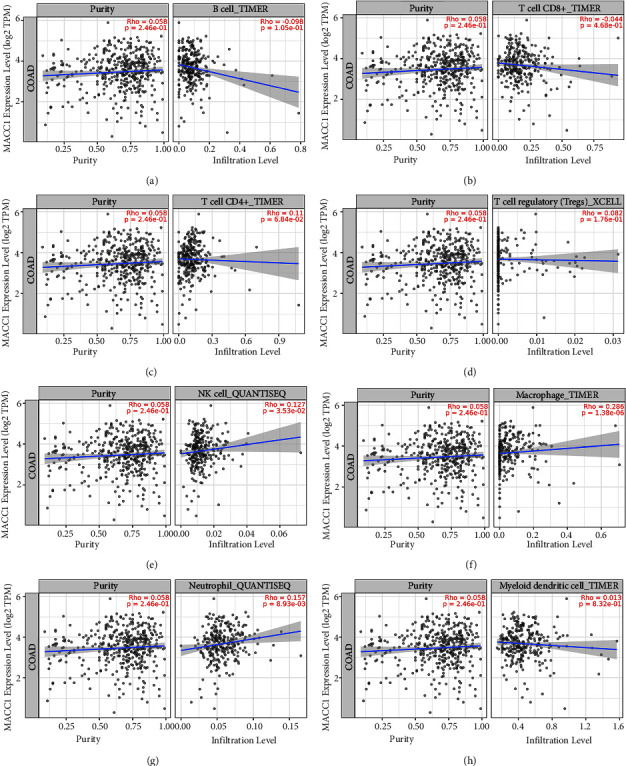
Correlation of *MACC*1 expression with immune cell infiltration level in COAD. The correlation of *MACC*1 expression level with *B* cell (a), CD8^+^*T* cell (b), CD4^+^*T* cell (c), tregs (d), NK cells (e), macrophage (f), neutrophil (g), and dendritic cell (h) infiltration level in COAD.

Since *MACC*1 has been reported to be involved in tumor immunity, the correlation between *MACC*1 mRNA expression and immune cell infiltration level was evaluated. *MACC*1 mRNA expression was found to be positively and significantly associated with the levels of NK cells, macrophages, and neutrophils, but not with the levels of *B* cells, CD8^+^*T* cells, CD4^+^*T* cells, Tregs, and dendritic cells in COAD (Figure 8). The potential role of *MACC*1 in tumor immunity was further assessed by evaluating the correlation between *MACC*1 mRNA expression and biomarkers of immune cells in COAD using the GEPIA2 database. *MACC*1 mRNA expression was positively correlated with biomarkers of NK cells (CD56), *M*1 macrophages (TLR2 and IRF5), *M*2 macrophages (CD206, CD115, and Dectin-1), neutrophils (CD11b, CEACAM8, ITGAM and MPO), and dendritic cells (NRP1, ITGAX and CD83) in COAD (Table 3). These findings suggest that *MACC*1 is involved in the immune regulation of COAD by regulating immune cell infiltration.

## 4. Discussion

Although *MACC*1 was shown to be a critical regulator and biomarker for progression and metastasis in over 20 types of cancer, including colon cancer, HCC, bladder cancer, and esophageal cancers [23–26], the mechanisms by which *MACC*1 is involved in the development and progression of COAD remain unclear. The present study analyzed the expression of *MACC*1 in 33 types of cancer using data from the TCGA and GTEx databases and found that *MACC*1 mRNA was more highly expressed in COAD than in corresponding normal colon tissues. The expression of *MACC*1 mRNA in COAD was subsequently validated by analyses of the GEPIA2 and CPTAC databases. Survival analysis indicated that high *MACC*1 mRNA expression in patients with COAD was associated with poor prognosis, which was consistent with previous findings [27]. *MACC*1 knockdown was also found to markedly inhibit cell proliferation, migration, invasion, colony formation, and tumorigenesis, both in vitro and in vivo, and to induce apoptosis in colorectal cancer (CRC) cells [28]. These findings suggested that *MACC*1 has an important role in CRC carcinogenesis and progression through the *β*-catenin signaling pathway and mesenchymal-epithelial transition [28]. Combined with the results of the present study, these findings demonstrate that *MACC*1 plays a carcinogenic role in COAD.

Non-coding RNAs (ncRNAs), including small microRNAs (miRNAs), lncRNAs, and circular RNAs (circRNAs), were shown to be involved in multiple biological processes in various types of cancer by directly or indirectly interfering with gene expression by inter-communication through the ceRNA mechanism [29–33]. Thus, upstream regulatory miRNAs of *MACC*1 could be predicted using the program starBasev2.0, which includes PITA, RNA22, miRmap, microT, miRanda, PicTar, and TargetScan[34]. Of the 33 miRNAs identified, most were found to be tumor suppressive in various types of cancers. For example, hsa-miR-18a-5p significantly reduced the hazard of dying in patients with CRC, regardless of tumor site [35], and low expression of miR-642a-5p was associated with a poor prognosis in patients with CRC. Moreover, miR-642a-5p was found to inhibit colon cancer cell migration, invasion, and EMT by targeting COL1A1 [36]. Overexpression of Neural Cell Adhesion Molecule 1 significantly inhibited the migration of ameloblastoma cells and was regulated by miR-141-3p [37]. In CRC, miR-186-5p acts as a cell cycle suppressor to inhibit tumor progression. Over-expression of miR-186-5p was found to significantly down-regulate the expression of SMAD6/7, resulting in decreased expression of CyclinD1 and c-Myc. Furthermore, over-expression of miR-186-5p promoted CRC cell apoptosis and inhibited their viability, proliferation, and migration [38]. Expression and correlation analyses showed that hsa-miR-642a-5p was the most likely tumor-suppressive miRNA targeting *MACC*1. No study to date, however, has assessed the role of hsa-miR-642a-5p targeting of *MACC*1 in CRC.

Based on the ceRNA hypothesis [39], the levels of expression of lncRNA and mRNA should correlate with each other. Thus, the potential lncRNAs associated with the hsa-miR-642a-5p/*MACC*1 axis in COAD should be highly expressed. Analysis using starBase v2.0 software predicted 113 lncRNAs that could be associated with the hsa-miR-642a-5p/*MACC*1 axis. Expression and correlation analyses identified ZFAS1 as the most likely upstream lncRNA. ZFAS1 lncRNA is a major protein regulator involved in various human cancers, including COAD. ZFAS1 has been shown to promote the proliferation, migration, and invasion of esophageal squamous cell carcinoma (ESCC) cells, and to inhibit their apoptosis by upregulating STAT3 and downregulating miR-124 [40]. ZFAS1 was also more highly expressed in glioma tissues and cells than in normal brain tissues and normal astrocyte HA cells. Moreover, high ZFAS1 expression was associated with poor prognosis among patients with glioma. Downregulation of ZFAS1 or upregulation of miR-1271-5p was found to inhibit the progression of glioma by enhancing the apoptosis and inhibiting the repressing proliferation, migration, and invasion of glioma cells [41]. The oncogenic activity of ZFAS1 and its significant upregulation, together with the elevated expression of DDX21 and POLR1B in CRC cells and tissues, further leads to poor clinical outcomes. Knockdown of ZFAS1 substantially suppressed CRC cell proliferation, invasion, and migration, and increased cell apoptosis [42]. ZFAS1 has also been shown to increase NOP58 and SNORD12C/78 expression in CRC cells and tissues [43]. Furthermore, ZFAS1 was found to promote the progression of CRC by competitively binding miR-150-5p, which plays a tumor suppressor role in CRC by targeting VEGFA [44]. Taken together, these findings suggest that ZFAS1 plays a carcinogenic role in various human cancers and that the ZFAS1/hsa-miR-642a-5p/*MACC*1 axis constitutes the potential regulatory pathway that promotes the progression of COAD.

Immune cell infiltration into tumors has been shown to affect the effectiveness of chemotherapy, radiotherapy, and immunotherapy, as well as the prognosis of cancer patients [45–47]. The present study showed that *MACC*1 upregulation increases the infiltration of various immune cells into COADs, including NK cells, macrophages, and neutrophils. Moreover, *MACC*1 expression showed significant positive correlations with biomarkers of NK cells, macrophages, neutrophils, and dendritic cells. These results suggest that *MACC*1 is associated with immune cell invasion into COAD. However, the present study has several limitations. The results of this study were based on bioinformatics analysis from various databases but were not validated experimentally. Confirmation of these findings requires in vivo and in vitro experiments.

**Figure 9:**
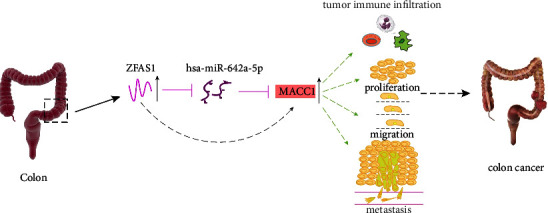
Model of the ZFAS1-hsa-miR-642a-5p-*MACC*1 axis and its potential roles in carcinogenesis of COAD.

In conclusion, this study we have demonstrated that *MACC*1 is overexpressed in several types of human cancers and that *MACC*1 overexpression correlates with poor prognosis and increased tumor immune cell infiltration in patients with COAD. The present study also identified the ZFAS1/hsa-miR-642a-5p axis (Figure 9) as the most likely upstream pathway of *MACC*1 in COAD. These results suggest that *MACC*1 participates in tumor immunity and progression of COAD through the ZFAS1/hsa-miR-642a-5p/*MACC*1 pathway.

## Figures and Tables

**Table 1:** Clinical characteristics of the COAD patients (TCGA).

Characteristic	Low expression of *MACC*1 (*n* = 239)	High expression of *MACC*1 (*n* = 239)	*p* value
*T* stage, *n* (%)			0.485
*T*1	8 (1.7%)	3 (0.6%)	
*T*2	40 (8.4%)	43 (9%)	
*T*3	161 (33.8%)	162 (34%)	
*N* stage, *n* (%)			0.081
*N*0	154 (32.2%)	130 (27.2%)	
*N*1	48 (10%)	60 (12.6%)	
*N*2	37 (7.7%)	49 (10.3%)	
*M* stage, *n* (%)			0.038^*∗*^
*M*0	184 (44.3%)	165 (39.8%)	
*M*1	25 (6%)	41 (9.9%)	
Pathologic stage, *n* (%)			0.019^*∗*^
Stage I	43 (9.2%)	38 (8.1%)	
Stage II	108 (23.1%)	79 (16.9%)	
Stage III	60 (12.8%)	73 (15.6%)	
Stage IV	25 (5.4%)	41 (8.8%)	
Gender, *n* (%)			0.410
Female	118 (24.7%)	108 (22.6%)	
Male	121 (25.3%)	131 (27.4%)	
Age, *n* (%)			0.780
≤65	99 (20.7%)	95 (19.9%)	
>65	140 (29.3%)	144 (30.1%)	
Age, median (IQR)	69 (59, 79)	69 (58, 76.5)	0.479
CEA level, *n* (%)			0.188
≤5	103 (34%)	93 (30.7%)	
>5	47 (15.5%)	60 (19.8%)	
Perineural invasion, *n* (%)			0.842
No	60 (33.1%)	75 (41.4%)	
Yes	19 (10.5%)	27 (14.9%)	
Lymphatic invasion, *n* (%)			0.972
No	136 (31.3%)	130 (30%)	
Yes	87 (20%)	81 (18.7%)	
OS event, *n* (%)			0.266
Alive	193 (40.4%)	182 (38.1%)	
Dead	46 (9.6%)	57 (11.9%)	
DSS event, *n* (%)			0.432
Alive	205 (44.4%)	193 (41.8%)	
Dead	29 (6.3%)	35 (7.6%)	

^*∗*^ *p* < 0.05.

**Table 2:** Correlation between lncRNAs and has-miR-642a-5p or lncRNAs and *MACC*1 in COAD determined by starBasev2.0 database.

lncRNA	miRNA	*R* value	*p* value
MIR4435-2HG	hsa-miR-642a-5p	0.011	8.19*E* − 01
LINC00511	hsa-miR-642a-5p	−0.024	6.05*E* − 01
MAFG-AS1	hsa-miR-642a-5p	−0.046	3.28*E* − 01
ZFAS1	hsa-miR-642a-5p	−0.185	7.75*E* − 05^*∗∗∗*^

*lncRNA*	*mRNA*	*R value*	*pvalue*
MIR4435-2HG	*MACC*1	0.226	7.08*E* − 07^*∗∗∗*^
LINC00511	*MACC*1	0.137	2.95*E* − 03^*∗∗*^
MAFG-AS1	*MACC*1	−0.163	3.82*E* − 04^*∗∗∗*^
ZFAS1	*MACC*1	0.178	1.03*E* − 04^*∗∗∗*^

^*∗*^ *p* < 0.05; ^*∗∗*^*p* < 0.01; ^*∗∗∗*^*p* < 0.001.

**Table 3:** Correlation between *MACC*1 and biomarkers of immune cells in COAD determined by GEPIA2 database.

Immune cell	Biomarker	*R* value	*p* value
*B* cell	CD19	−0.069	0.25
	CD79A	−0.096	0.11
	CD79B	−0.12	0.046^*∗*^

CD8^+^*T* cell	CD8A	−0.18	0.003^*∗∗*^
	CD8B	−0.091	0.13

CD4^+^*T* cell	CD4	0.094	0.12

*M*1 macrophage	CD80	0.059	0.33
	CD86	0.067	0.27
	TLR2	0.17	0.0044^*∗∗*^
	IRF5	0.19	0.0013^*∗∗*^
	PTGS2	0.046	0.44

*M*2 macrophage	CD163	0.053	0.38
	MS4A4A	0.059	0.33
	CD206	0.14	0.017^*∗*^
	CD115	0.15	0.016^*∗*^
	CD301	0.019	0.75
	Dectin-1	0.12	0.04^*∗*^
	ARG1	0.065	0.28

Neutrophil	CD11b	0.16	0.0089^*∗∗*^
	CEACAM8	0.18	0.0022^*∗∗*^
	ITGAM	0.16	0.0089^*∗∗*^
	MPO	0.13	0.028^*∗*^
	CCR7	−0.02	0.75

Dendritic cell	HLA-DPB1	−0.058	0.34
	HLA-DQB1	−0.097	0.11
	HLA-DPA1	−0.042	0.49
	CD1C	0.052	0.39
	NRP1	0.19	0.0015^*∗∗*^
	ITGAX	0.15	0.012^*∗*^
	CD83	0.19	0.0015^*∗∗*^

These results are statistically significant. ^*∗*^*p* < 0.05; ^*∗∗*^*p* < 0.01; ^*∗∗∗*^*p* < 0.001.

## Data Availability

The datasets for this study can be found in these websites: (1) TCGA database https://portal.gdc.cancer.gov/, (2) Genotype-Tissue Expression Project (GTEx) database https://www.genome.gov/, (3) GEPIA2 database https://gepia2.cancer-pku.cn/#index, (4) Clinical Proteomic Tumor Analysis Consortium (CPTAC) https://proteomics.cancer.gov/programs/cptac, (5) UALCAN database https://ualcan.path.uab.edu, (6) starBasev2.0 database analysis https://starbase.sysu.edu.cn/,and (7) TIMER2.0 database analysis https://timer.cistrome.org/.

## Supplementary Materials

Supplementary Materials Supplementary Table S1. Identification of upstream potential miRNAs of *MACC*1 in COAD. The upstream potential miRNAs of *MACC*1 in COAD were identified in the starBasev2.0 database.
