# Supplementary material for: MACC1 Correlates with Tumor Progression and Immune Cell Infiltration of Colon Adenocarcinoma and is Regulated by the lncRNA ZFAS1/miR-642a-5p Axis
Source: J Oncol. 2022 Dec 12;2022:8179208. doi: 10.1155/2022/8179208 (PMC9763013; doi:10.1155/2022/8179208)
Supplement: Supplementary Materials — Supplementary Table S1. Identification of upstream potential miRNAs of MACC1 in COAD. The upstream potential miRNAs of MACC1 in COAD were identified in the starBasev2.0 database. [file 8179208.f1.docx]

Table S1. Identifition of upstream potential miRNAs of MACC1 in COAD

|  |  | **COAD** | |
| --- | --- | --- | --- |
| **Gene** | **miRNA** | **R-value** | **P-value** |
| **MACC1** | **hsa-miR-18a-5p** | **-0.007** | **8.81E-01** |
| **MACC1** | **hsa-miR-141-3p** | **-0.138** | **3.25E-03** |
| **MACC1** | **hsa-miR-142-5p** | **-0.283** | **9.19E-10** |
| **MACC1** | **hsa-miR-145-5p** | **0.109** | **2.07E-02** |
| **MACC1** | **hsa-miR-126-5p** | **-0.219** | **2.64E-06** |
| **MACC1** | **hsa-miR-186-5p** | **-0.16** | **6.81E-04** |
| **MACC1** | **hsa-miR-155-5p** | **-0.168** | **3.38E-04** |
| **MACC1** | **hsa-miR-200a-3p** | **-0.087** | **6.54E-02** |
| **MACC1** | **hsa-miR-374a-5p** | **0.223** | **1.86E-06** |
| **MACC1** | **hsa-miR-380-3p** | **-0.053** | **2.66E-01** |
| **MACC1** | **hsa-miR-337-3p** | **0.011** | **8.23E-01** |
| **MACC1** | **hsa-miR-323a-3p** | **-0.059** | **2.09E-01** |
| **MACC1** | **hsa-miR-18b-5p** | **0.122** | **9.64E-03** |
| **MACC1** | **hsa-miR-329-3p** | **0.132** | **5.03E-03** |
| **MACC1** | **hsa-miR-410-3p** | **0.212** | **5.59E-06** |
| **MACC1** | **hsa-miR-494-3p** | **0.156** | **9.24E-04** |
| **MACC1** | **hsa-miR-498** | **0.128** | **6.75E-03** |
| **MACC1** | **hsa-miR-579-3p** | **0.055** | **2.48E-01** |
| **MACC1** | **hsa-miR-642a-5p** | **-0.226** | **1.26E-06** |
| **MACC1** | **hsa-miR-362-3p** | **0.037** | **4.40E-01** |
| **MACC1** | **hsa-miR-379-3p** | **0.102** | **3.12E-02** |
| **MACC1** | **hsa-miR-340-5p** | **0.151** | **1.27E-03** |
| **MACC1** | **hsa-miR-411-3p** | **0.195** | **3.09E-05** |
| **MACC1** | **hsa-miR-889-3p** | **0.07** | **1.36E-01** |
| **MACC1** | **hsa-miR-885-5p** | **-0.033** | **4.82E-01** |
| **MACC1** | **hsa-miR-877-5p** | **0.142** | **2.60E-03** |
| **MACC1** | **hsa-miR-374b-5p** | **0.199** | **2.12E-05** |
| **MACC1** | **hsa-miR-1185-5p** | **-0.019** | **6.87E-01** |
| **MACC1** | **hsa-miR-3121-3p** | **-0.035** | **4.56E-01** |
| **MACC1** | **hsa-miR-642b-3p** | **0.041** | **3.82E-01** |
| **MACC1** | **hsa-miR-642a-3p** | **0.016** | **7.35E-01** |
| **MACC1** | **hsa-miR-5195-3p** | **0.051** | **2.85E-01** |
| **MACC1** | **hsa-miR-5590-3p** | **0** | **1.00E+00** |
